# Socioeconomic differences in prevalence and patterns of non-communicable disease multimorbidity and its impact on depression in rural Southwest China

**DOI:** 10.1016/j.pmedr.2025.103235

**Published:** 2025-09-04

**Authors:** Lan Liu, Guo-hui Li, Xia Wu, Bo Lv, Allison Rabkin Golden, Ling-Li Dong, Le Cai

**Affiliations:** a Yunnan Provincial Key Laboratory of Public Health and Biosafety & School of Public Health, Kunming Medical University, Kunming 650500, China; b The Second Affiliated Hospital of Kunming Medical University, China; c The First Affiliated Hospital of Kunming Medical University, China

**Keywords:** Chronic non-communicable diseases, Multimorbidity, Socioeconomic status, Depression symptoms, China

## Abstract

**Objective:**

This study aimed to uncover socioeconomic variations in the prevalence and patterns of noncommunicable disease (NCD) multimorbidity including seven common chronic conditions, and to investigate its association with depression symptoms.

**Methods:**

A cross-sectional survey was conducted including 7509 adults aged ≥35 years in rural Yunnan Province, China from 2023 to 2024. Association rule mining was used to identify the NCD multimorbidity patterns of seven common chronic conditions. Multivariable logistic regression analysis was employed to examine the relationship between NCD multimorbidity and depression symptoms.

**Results:**

The prevalence of NCD multimorbidity was 18.8 %. NCD multimorbidity was more prevalent among participants with lower education, lower annual household income, and better access to medical care (*P* < 0.05). NCD multimorbidity and their patterns of chronic obstructive pulmonary disease + asthma, diabetes + coronary heart disease, hypertension + stroke, and hypertension + coronary heart disease + diabetes were associated with a greater risk of depression symptoms (*P* < 0.05).

**Conclusions:**

There are socioeconomic disparities in prevalence and patterns of NCD multimorbidity across rural southwest China. NCD multimorbidity was positively associated with depressive symptoms. Future measures to prevention NCD multimorbidity should be strengthened and targeted at lower socioeconomic populations, with greater focus on managing depression in NCD multimorbid patients.

## Highlights

• Socioeconomic disparities in prevalence and patterns of multimorbidity were found.
• Hypertension + diabetes was the most prevalent multimorbidity pattern.
• Multimorbidity was positively associated with depression symptoms.

## 1 Introduction

Non-communicable diseases (NCDs) are a formidable global health challenge, contributing to approximately 41 million deaths annually, with 77 % of these deaths occurring in low- and middle-income countries (LMICs) (Mohan et al., 2023). Multimorbidity, defined as the simultaneous presence of two or more chronic conditions within an individual, has emerged as a critical concern in the context of NCDs (Skou et al., 2022). China's large and rapidly aging population is contributing to a mounting epidemic of multimorbidity: a national study indicated the prevalence of multimorbidity among the elderly rose from 15.60 % in 1998 to 30.76 % in 2018, with a significant rise noted in southern China (Chen et al., 2021).

Multimorbidity is associated with a spectrum of adverse health outcomes, including reduced functional capacity, increased disability, and higher mortality rates (Skou et al., 2022). However, there remains a pressing need for further investigation into its implications for mental disorders to better inform care. Depression, among the most important and prevalent mental disorders, is closely linked to multimorbidity. A meta-analysis of 40 studies revealed that people with multimorbidity had 2–3 times greater risk of depression compared to those without any chronic diseases (Read et al., 2017). However, the precise relationship between specific patterns of multimorbidity and depression is not clear, with few studies exploring this association.

Socioeconomic factors are increasingly recognized as critical determinants of health outcomes. Research suggests that people with lower socioeconomic status (SES) are more likely to develop multimorbidity (Violan et al., 2014). Likewise, the distribution of multimorbidity patterns is also affected by socioeconomic differences across populations (e.g., education, income, and ethnicity) (Álvarez-Gálvez et al., 2023). In several studies, low income and low educational attainment have been linked with increased odds of cardiometabolic, musculoskeletal, and respiratory issues (Álvarez-Gálvez et al., 2023; Álvarez-Gálvez et al., 2022). Furthermore, ethnic disparities contribute to variations in the prevalence of certain chronic disease combinations (Alshakhs et al., 2022; Kalgotra et al., 2020). To date, there is little research addressing the influence of socioeconomic determinants on both the prevalence and patterns of multimorbidity in rural China.

In recent years, research has uncovered the diverse multimorbidity patterns of chronic diseases with analytical techniques including factor analysis, cluster analysis, and simple prevalence (Skou et al., 2022; Álvarez-Gálvez et al., 2023). Association rule mining (ARM) provides another valuable tool for exploring hidden patterns or associations within large databases, generating association rules with high confidence and relevance across a large set of items (Lee et al., 2020; Agrawal et al., 1993). Previous research conducted in other countries using ARM has identified prevalent and strongly associated multimorbidity patterns, such as “hypertension and high cholesterol,” and “hypertension, arthritis and diabetes” (Lee et al., 2020; Zheng et al., 2022). However, these findings might not be directly applicable to the Chinese population due to variations in the disease spectrum and other contextual factors.

Thus, this study aims to use ARM to examine the prevalence and patterns of seven common NCDs' multimorbidity (hypertension, diabetes, hyperlipidemia, coronary heart disease (CHD), stroke, chronic obstructive pulmonary disease (COPD), and asthma) and explore how they differ by socioeconomic factors (including ethnicity, level of education, annual household income, and access to medical services), as well as investigate the relationship between NCD multimorbidity and its specific patterns with depression symptoms in rural southwest China.

## 2 Methods

### 2.1 Study area and population

The present study was conducted in three rural areas of Yunnan Province from 2023 to 2024 and employed a community-based, cross-sectional design. Located in the southwest of China, Yunnan is one of the least developed provinces in the country. Half of its 47.2 million residents reside in rural areas.

In this study, Yunnan's 129 counties were divided into three categories based on their economic development level (GDP per capita): low-, middle-, and high-income. One county was randomly chosen from each category for a total of three counties. A three-stage stratified random sampling procedure was employed to select participants. First, each selected county was divided into two categories (high and low) according to per capita GDP. From each category, one township was randomly selected, for a total of six townships. Second, probability proportional to size method was used to select three villages from each of the six townships. Finally, simple random sampling was conducted to select the sample subjects from a village based on a list of individuals aged ≥35 years obtained from each selected village's leadership committee.

### 2.2 Data collection and measurement

Twenty medical students from Kunming Medical University were trained as interviewers for data collection. All participants who gave informed consent were then personally interviewed by one of the interviewers using a structured questionnaire. The questionnaire included demographic and socioeconomic questions, including sex, age, ethnicity, level of education, annual household income, and walking time to the nearest village clinic.

Three consecutive blood pressure measurements were also taken from each participant at 5 min intervals (Pickering et al., 2005). The average of the three readings of blood pressure was recorded. Fasting blood glucose (FBG) and post-bronchodilator pulmonary function tests (including pre- and post-bronchodilator forced expiratory volume in one second (FEV1) and forced vital capacity (FVC)) were performed by physicians from an affiliated hospital of Kunming Medical University.

The Zung's Self-rating Depression Scale (SDS) was used to assess the presence of depressive symptoms. The scale consists of 20 items covering affective, psychological, and somatic symptoms associated with depression. It has been validated in the Chinese population and shown to have satisfactory psychometric properties (Lee et al., 1994). For each item, participants were asked to choose the statement that best described their symptomatology in the past week, using a four-point Likert scale ranging from one to four. The total raw score of the SDS was then converted to an SDS index score by multiplying the raw score by 1.25, resulting in the index score ranging from 25 to 100.

### 2.3 Definitions

Hypertension was defined as mean systolic blood pressure ≥ 140 mmHg, mean diastolic blood pressure ≥ 90 mmHg, and/or self-reported use of antihypertensive medications (Pickering et al., 2005). Diabetes was defined as FBG ≥ 7.0 mmol/l (126 mg/dl), self-reported diagnosis of diabetes by a health professional, and/or use of antidiabetic medication (*Diabetes Care*, 2003). COPD was defined according to the Global Initiative for Chronic Obstructive Lung Disease (GOLD) guidelines as post-bronchodilator FEV1/FVC < 70 % (Vestbo et al., 2013). Hyperlipidemia, CHD, stroke, and asthma were defined by self-reported history of physician diagnosis. Multimorbidity was defined as the co-existence of two or more chronic conditions in an individual among the seven chronic diseases under investigation in this study. Respondents with an SDS index score of 53 or higher were classified as having depression symptoms.

China, a multi-ethnic nation, officially recognizes the Han as the majority and 55 ethnic minorities. In this study, ethnicity was categorized into Han majority and minority ethnic groups. The minority ethnic groups included populations whose religious, cultural, or linguistic backgrounds differed from the Han ethnic majority. Illiteracy was defined as the inability to read or write a short and simple statement about daily life. Educational level was classified into two categories: illiterate and primary (grades 1–6) or higher. Annual household income was divided into low and high groupings based on the median value. Access to medical services was classified into two groups, defined by self-reported walking time from a participant's home to the nearest village clinic, with a cut-off of 30 min: poor (>30 min.), good (≤30 min.).

### 2.4 Ethical approval

This study was approved by the Ethics Committee of Kunming Medical University (approval number: KMMU2020MEC031) prior to the commencement of research.

### 2.5 Statistical analysis

Data were analyzed with descriptive statistics, chi-squared tests, *t*-tests, ARM, and multivariable logistic regression. Categorical variables were summarized by calculating counts and percentages. Continuous variables were presented as mean ± standard deviation (SD). Differences between groups were compared by *t*-test for continuous variables while $\chi^{2}$ tests of independence were used for categorical variables.

ARM based on the Apriori algorithm was employed to identify the critical chronic condition combinations that satisfied the minimum threshold of measurement indicators. Three main indicators–support, confidence, and lift–were used to select frequent, reliable, and logical rules. Support indicates how often the rules (chronic disease combinations, in this study) appear in the dataset. Confidence refers to the frequency with which a rule is found to be true and measures the reliability of the rule. Lift is calculated as a rule's observed support to the support of the individual chronic disease in the rule. Lift assesses the importance of rules, with a higher lift value (>1) implying a stronger association, and thus being the main measure of significance in the study. The thresholds for association rules were set as follows: support >0.1 %, confidence >20 %, and lift >1. All association rules were sorted by lifts, and the top 10 association rules with larger lifts were described.

Multivariable logistic regression was used to examine the association between NCD multimorbidity and its patterns with depression. Adjusted odds ratios (AOR) and 95 % confidence intervals (CI) were reported. All data analyses were performed using R software, version 4.2.1. All statistical significance decisions were based on two-tailed *P* values of <0.05.

## 3 Results

In the present study, a total of 7800 individuals aged ≥35 years were invited to participate and 7509 consented to participate. Table 1 presents the demographic characteristics and mean values of blood pressure, FBG, and spirometry of the study participants. Participants in the study included 3720 males and 3789 females. Among the study participants, 43.4 % were ethnic minorities and 24.3 % were illiterate.

**Table 1:** Demographic characteristics and mean values of blood pressure, fasting blood glucose, and spirometry among participants in rural Yunnan Province, 2023–2024.

Characteristic n (%) / mean ± SD	Male (*n* = 3720)	Female (*n* = 3789)	All (*n* = 7509)
Age			
35–44 years	271(7.3)	315(8.3)	586(7.8)
45–59 years	1352(36.3)	1421(37.5)	2773(36.9)
≥60 years	2097(56.4)	2053(54.2)	4150(55.3)
Ethnicity			
Han	2214(59.5)	2143(56.6)	4357(58.0)
Minority	1506(40.5)	1646(43.4)	3152(42.0)
Education			
Illiterate	296(8.0)	922(24.3) ⁎⁎	1218(16.2)
Primary (grade 1–6) or higher	3424(92.0)	2867(75.7)	6291(83.8)
Annual household income			
Low	1795(48.2)	1927(51.8) ⁎	3722(49.6)
High	1925(50.2)	1862(49.2)	3787(50.4)
Access to medical services			
Poor	1081(29.1)	1177(31.1)	2258(30.1)
Good	2639(70.9)	2612(68.9)	5251(69.9)
Systolic blood pressure (mm Hg)	136.3 ± 20.3	137.2 ± 22.2	136.7 ± 21.3
Diastolic blood pressure (mm Hg)	84.6 ± 12.1	83.8 ± 11.6 ⁎⁎	84.2 ± 11.9
FBG (mmol/l)	6.9 ± 2.6	6.7 ± 2.3 ⁎	6.8 ± 2.5
Spirometry			
FEV1, L	2.1 ± 0.6	1.5 ± 0.4 ⁎⁎	1.8 ± 0.6
FVC, L	2.6 ± 0.7	1.9 ± 0.5 ⁎⁎	2.3 ± 0.7
FEV1/FVC	79.9 ± 6.9	80.2 ± 6.5 ⁎	80.1 ± 6.9

⁎ *p*-value is based on chi-square test or *t*-test; Abbreviations: FBG Fasting blood glucose, FEV1 forced expiratory volume in one second, FVC: forced vital capacity, SD standard deviation.

Table 2 shows the prevalence of seven chronic conditions and depression symptoms by sex and age. The overall prevalence of hypertension, diabetes, hyperlipidemia, CHD, stroke, COPD, asthma, and depression symptoms was 57.6 %, 10.6 %, 5.8 %, 6.2 %, 3.4 %, 5.1 %, 0.9 %, and 4.0 %, respectively. Males had significantly higher prevalence of COPD than females, whereas females had higher prevalence of hyperlipidemia and depression symptoms than males (*P* < 0.05). The prevalence of seven chronic conditions and depression increased with age, peaking in the ≥60 years age group (*P* < 0.05).

**Table 2:** Age- and sex-stratified prevalence of seven chronic diseases and depression symptoms among participants in rural Yunnan Province, 2023–2024.

Characteristic n (%)	Hypertension	Diabetes	Hyperlipidemia	CHD	Stroke	COPD	Asthma	Depression symptoms
Sex								
Male	2112(56.8)	404(10.9)	195(5.2)	250(6.7)	134(3.6)	221(5.9) **	32(0.9)	97(2.6)
Female	2189(57.8)	391(10.3)	243(6.4) *	216(5.7)	120(3.2)	161(4.2)	37(1.0)	201(5.3) **
Age								
35–44 years	178(30.4)	27(4.6)	18(3.1)	4(0.7)	1(0.2)	6(1.0)	1(0.2)	10(1.7)
45–59 years	1327(47.9)	243(8.8)	168(6.1)	79(2.8)	46(1.7)	59(2.1)	13(0.5)	67(2.4)
≥60 years	2796(67.4) **	525(12.7) **	252(6.1) *	383(9.2) **	207(5.0) **	317(7.6) **	55(1.3) **	221(5.3) **
All	4301(57.3)	795(10.6)	438(5.8)	466(6.2)	254(3.4)	382(5.1)	69(0.9)	298(4.0)

⁎*p-*values were obtained using the chi-square test; Abbreviations: CHD coronary heart disease, COPD chronic obstructive pulmonary disease.

The association rules for seven chronic NCDs according to lift value are shown in Table 3. The NCD combination of COPD + asthma had the highest lift, indicating that the probability of these two conditions occurring together was 10.3 times higher than in isolation. Furthermore, hypertension was identified as the most common disease in these patterns, appearing in seven out of the ten rules and closely associated with diabetes, hyperlipidemia, CHD, and stroke.

**Table 3:** Top 10 association rules in order of lift for binary and ternary multimorbidity patterns among participants in rural Yunnan Province, 2023–2024.

Rules	Support (%)	Confidence (%)	Lift
Binary multimorbidity patterns			
COPD +Asthma	1.0	52.2	10.3
Diabetes + Hyperlipidemia	5.8	31.1	2.9
Diabetes + CHD	6.2	22.5	2.1
Hypertension + Stroke	3.4	84.3	1.5
Hypertension + Diabetes	10.6	76.1	1.3
Hypertension + Hyperlipidemia	5.8	74.4	1.3
Hypertension + CHD	6.2	72.8	1.3
Ternary multimorbidity patters			
Hyperlipidemia + Hypertension + Diabetes	4.3	35.9	3.4
Hypertension + CHD + Diabetes	4.5	26.5	2.5
Hyperlipidemia + Hypertension + CHD	1.1	85.9	1.5

Abbreviations: CHD coronary heart disease, COPD chronic obstructive pulmonary disease; Support refers to the relative frequency of a rule in a dataset, indicating how often the rule is observed; Confidence measures the reliability of a rule, representing the conditional probability that the consequent chronic diseases (e.g., hypertension and CHD) are present given the antecedent chronic disease (e.g., diabetes); Lift quantifies the strength of the association rule, calculated as a rule's observed support to the expected support of the individual chronic disease.

Table 4 displays the prevalence of seven chronic NCDs' multimorbidity and their patterns by socioeconomic characteristics. The overall prevalence of NCD multimorbidity was 18.8 % in the study population. Hypertension + diabetes (8.1 %) was the most common multimorbidity pattern. Participants with lower education, lower annual household income, and good access to medical services had significantly higher prevalence of NCD multimorbidity than their counterparts (*P* < 0.05). Prevalence of hypertension + diabetes decreased as levels of education and annual household income increased, whereas a higher prevalence of hypertension combined with hyperlipidemia and diabetes was observed among those with good access to medical services (*P* < 0.05).

**Table 4:** Prevalence and patterns of multimorbidity by socioeconomic characteristics among participants in rural Yunnan Province, 2023–2024.

Characteristic n (%)	Multimorbidity	COPD + Asthma	Diabetes + Hyperlipidemia	Diabetes + CHD	Hypertension + Stroke	Hypertension + Diabetes	Hypertension + Hyperlipidemia	Hypertension + CHD	Hyperlipidemia + Hypertension + Diabetes	Hypertension + CHD + Diabetes	Hyperlipidemia + Hypertension + CHD
Ethnicity											
Han	847(19.4)	23(0.5)	90(2.1)	58(1.3)	139(3.2) *	349(8.0)	207(4.8) *	216(5.0) *	76(1.7)	48(1.1)	39(0.9)
Minority	561(17.8)	13(0.4)	46(1.5)	47(1.5)	75(2.4)	256(8.1)	119(3.8)	123(3.9)	41(1.3)	42(1.3)	22(0.7)
Education											
Illiterate	253(20.8) *	9(0.7)	25(2.1)	15(1.2)	41(3.4)	116(9.5) *	48(3.9)	58(4.8)	23(1.9)	12(1.2)	6(0.5)
Primary (grade 1–6) or higher	1155(18.4)	27(0.4)	111(1.8)	90(1.4)	173(2.7)	489(7.8)	278(4.4)	281(4.5)	94(1.5)	78(1.2)	55(0.9)
Annual household income											
Low	733(19.7) *	14(0.4)	65(1.7)	48(1.3)	117(3.1)	323(8.7) *	151(4.1)	177(4.8)	52(1.4)	42(1.1)	29(0.8)
High	675(17.8)	22(0.6)	71(1.9)	57(1.5)	97(2.6)	282(7.4)	175(4.6)	162(4.3)	65(1.7)	48(1.3)	32(0.8)
Access to medical services											
Poor	379(16.8)	11(0.5)	38(1.7)	30(1.3)	74(3.3)	153(6.8)	79(3.5)	104(4.6)	30(1.3)	25(1.1)	16(0.7)
Good	1029(19.6) *	25(0.5)	98(1.9)	75(1.4)	140(2.7)	452(8.6) **	247(4.7) *	235(4.5)	87(1.7)	65(1.2)	45(0.9)
	1408(18.8)	36(0.5)	136(1.8)	105(1.4)	214(2.8)	605(8.1)	326(4.3)	339(4.5)	117(1.6)	90(1.2)	61(0.8)

⁎*p-*values were obtained using the chi-square test; Abbreviations: CHD coronary heart disease, COPD chronic obstructive pulmonary disease.

Table 5 indicates the results of multivariable logistic regression analysis for prevalence of depression symptoms by NCD multimorbidity and their patterns. After adjusting for sex, age, ethnicity, education, household income, and access to medical services, participants with NCD multimorbidity had double the odds of developing depression as compared to those without multimorbidity (OR: 2.07[1.57–2.72]). Among the ten NCD multimorbidity combinations, participants with COPD + asthma (OR: 3.34[1.29–8.66]), diabetes + CHD (OR: 1.87[1.12–2.90]), hypertension + stroke (OR: 2.75[1.73–4.39]), and hypertension + CHD+ diabetes (OR: 2.91[1.44–5.56]) were associated with having a greater risk of suffering from depression symptoms (*P*<0.05).

**Table 5:** Odds ratios and 95 % confidence intervals for prevalence of depression symptoms by multimorbidity and its patterns among participants in rural Yunnan Province, 2023–2024.

Variable	Depression symptoms (reference: no)	
	Adjusted odds ratio (AOR) a	95 % CI
Multimorbidity (reference: no)	2.07	(1.57, 2.72)
COPD+ Asthma (reference: no)	3.34	(1.29, 8.66)
Diabetes + Hyperlipidemia (reference: no)	1.28	(0.51, 3.22)
Diabetes + CHD (reference: no)	1.87	(1.21, 2.90)
Hypertension + Stroke (reference: no)	2.75	(1.73, 4.39)
Hypertension + Diabetes (reference: no)	0.89	(0.53, 1.50)
Hypertension + Hyperlipidemia (reference: no)	0.62	(0.32, 1.22)
Hypertension + CHD (reference: no)	1.51	(0.90, 2.52)
Hyperlipidemia + Hypertension + Diabetes (reference: no)	0.93	(0.42, 2.07)
Hypertension + CHD + Diabetes (reference: no)	2.91	(1.44, 5.56)
Hyperlipidemia + Hypertension + CHD (reference: no)	1.00	(0.31, 3.21)

a Adjusted for sex, age, ethnicity, education, household income, and access to medical services; Abbreviations: CHD coronary heart disease, COPD chronic obstructive pulmonary disease, AOR adjusted odds ratios, CI Confidence interval.

## 4 Discussion

The findings indicate that both the prevalence and specific patterns of seven chronic NCDs' multimorbidity were independently associated with socioeconomic factors in rural southwest China, and chronic NCD multimorbidity and its certain patterns were positively associated with depression symptoms.

In accordance with previous epidemiological studies conducted in China (Zhang et al., 2022), and many other countries, including Finland, Russia, and South Africa (Garin et al., 2016), our study revealed hypertension was the most prevalent chronic disease in the surveyed population. Moreover, the prevalence of hypertension (57.3 %), diabetes (10.6 %), stroke (3.4 %), and CHD (6.2 %) was greater than reported in geographically or economically similar countries, including Brazil and India (Pereira et al., 2023; Gummidi et al., 2023), while prevalence of hyperlipidemia (5.8 %) and asthma (0.9 %) were comparatively lower than observed in these countries. The underlying determinants contributing to these observed disparities in epidemiological profiles warrant further exploration. These findings also underscore the necessity for context-specific strategies to effectively manage the burden of prevalent chronic diseases, like hypertension, CHD, and diabetes, in rural China.

This study also revealed sex differences in chronic disease distributions, aligning with results from previous studies in LMICs (Pereira et al., 2023; Gummidi et al., 2023). Specifically, males were more likely to suffer from COPD than females, whereas females exhibited a higher prevalence of hyperlipidemia compared to males. These differences were likely influenced by both physiological variances as well as differing lifestyle habits by sex (Le et al., 2023). Notably, all seven chronic conditions showed an increase in prevalence with advancing age, highlighting a concerning trend amid the rapidly aging population in China. The findings in this way suggest that developing both sex-specific and age-specific strategies for chronic disease prevention is crucial to promoting better health outcomes in rural populations.

Overall, the prevalence of the seven NCDs' multimorbidity in rural southwest China was 18.8 %, a relatively low rate compared to a recent systematic review reporting a prevalence range of 1.5 %–90.5 % among Chinese adults (Zhang et al., 2022). Nevertheless, the prevalence of multimorbidity varies widely across countries. A Nepalese study investigating six chronic conditions estimated a multimorbidity prevalence of 13.96 % (Dhungana et al., 2021), whereas a Canadian study reported a prevalence of 22.20 % (including eight NCDs) (Xiao et al., 2024). In the United States, the prevalence of multimorbidity was 27.2 % (involving ten NCDs) among the adult population (Boersma et al., 2020); in Kuwait, the rate was 27.4 % (involving twelve conditions) (Saoud et al., 2024). This discrepancy in NCD multimorbidity prevalence may be heavily influenced by the number and types of chronic conditions included and analyzed (Ho et al., 2021). While the prevalence rate of NCD multimorbidity has been inconsistent, it remains a formidable public health challenge for rural Yunnan communities with limited health resources.

The present study also indicated that individuals with low educational attainment and low income are more likely to have NCD multimorbidity. This inverse relationship between education levels, income, and the prevalence of NCD multimorbidity is in line with previous research (Xiao et al., 2024; Geng et al., 2023). Lower SES increases the risk of developing NCD multimorbidity, possibly due to its correlation with lower health literacy, a higher likelihood of engaging in unhealthy behaviors such as smoking and alcohol consumption, and/or less healthy diets (Allen et al., 2017). However, access to medical services was positively associated with the prevalence of NCD multimorbidity. This may result from the fact that better access to healthcare provides patients with more opportunities to obtain a diagnosis and report their diseases. Thus, this outcome highlights an urgent need to improve the prevention and management of NCD multimorbidity for those with low SES.

In our study, the results of ARM analysis found that COPD and asthma were the most related dyad NCDs combination. This finding is in line with previous studies in both clinical and community settings, suggesting shared risk factors, pathological mechanisms, and clinical characteristics between these respiratory diseases (Garin et al., 2016; Slats and Taube, 2016). Furthermore, the ranked lift also indicated strong association rules within cardiometabolic diseases, including dyad NCDs combinations of hypertension with stroke, diabetes, and hyperlipidemia, as well as triad NCDs combinations of hypertension, CHD, and diabetes. Indeed, the potential interactions between cardiometabolic diseases have been well documented (Li et al., 2022), also in line with our results. In this way, improved management of a single chronic disease in these high-lift combinations may have implications for preventing other associated diseases, thereby alleviating the burden of NCD multimorbidity.

The present study additionally uncovered that the most common NCD multimorbidity pattern was hypertension and diabetes. This result is similar to prior observations using ARM (Lee et al., 2020; Zheng et al., 2022). Additionally, there is growing evidence of socioeconomic disparities in NCD multimorbidity patterns (Álvarez-Gálvez et al., 2023; Álvarez-Gálvez et al., 2022), which were evident for certain NCD combinations observed in this study. Specifically, lower education levels and lower annual household income were associated with higher rates of hypertension and diabetes multimorbidity, a trend consistent with other research (Álvarez-Gálvez et al., 2023; Xie et al., 2019). Han participants exhibited a higher prevalence of hypertension combined with stroke, CHD, and diabetes than ethnic minority groups. This can be partly explained by higher rates of cardiovascular disease risk factors (e.g., obesity and physical inactivity) among Han participants (Le et al., 2023; Huang et al., 2024). However, poor access to medical services was linked to decreased hypertension combined with hyperlipidemia and diabetes, potentially in part due to underdiagnosis of these conditions.

The prevalence of depression symptoms in this study (4.0 %) was lower than previously measured rates in China (11.27 %) (Huang et al., 2021), as well as rates in other countries (5.2 % in Ghana, 9.3 % in Mexico, and 7.7 % in the United States) (Zheng et al., 2022; Arokiasamy et al., 2015). Furthermore, accumulating evidence suggests that multimorbidity can increase the risk of depression (Read et al., 2017). Similarly, our results identified that individuals with NCD multimorbidity had a more than two-fold higher risk of developing depression than those without multimorbidity. In addition, the specific dyad NCDs combinations - COPD and asthma, diabetes and CHD, hypertension and stroke – as well as the triad NCDs combination of hypertension, CHD, and diabetes, were linked to an increased risk of depression. This finding aligns with previous studies indicating respiratory, cardiovascular, and metabolic dysfunction affects mental health (Wang et al., 2025). More attention is needed on screening and treating depressive symptoms among NCD multimorbid individuals.

The present study was limited in several ways. First, its cross-sectional design precludes establishing causal or temporal relationships. Second, reliance on self-reported data for identifying hyperlipidemia, CHD, stroke, and asthma may have led to an underestimation of their prevalence, given the potential for individuals lacking access to healthcare services to be unaware of their conditions. Third, the measurement of multimorbidity was restricted to seven common chronic NCDs; the prevalence of multimorbidity in our study may be underestimated. Future inclusion of a broader range of diseases could improve the validity of prevalence estimates. Fourth, while the SDS-20 scale has been widely utilized in epidemiological research on depression, discrepancies may exist between self-rated measures and clinician-rated assessments. Finally, the present results were based on a random sampling of three counties, which limits their generalizability to the entire province.

## 5 Conclusion

The findings indicate there are significant socioeconomic differences in both the prevalence and specific patterns of NCDs multimorbidity. This study also demonstrated that NCDs multimorbidity, and certain patterns of multimorbidity, were positively associated with depression symptoms. Future measures to prevention NCDs multimorbidity should be strengthened and targeted at lower socioeconomic populations, with greater focus on managing depression in NCDs multimorbid patients.

## Availability of data and materials

The datasets used and/or analyzed during the current study are available from the corresponding author upon reasonable request.

## CRediT authorship contribution statement

**Lan Liu:** Writing – original draft, Visualization, Validation, Investigation, Formal analysis, Data curation. **Guo-hui Li:** Validation, Investigation, Writing – review & editing. **Xia Wu:** Validation, Investigation, Writing – review & editing. **Bo Lv:** Investigation, Conceptualization, Writing – review & editing. **Allison Rabkin Golden:** Writing – review & editing, Methodology. **Ling-Li Dong:** Writing – review & editing, Writing – original draft, Supervision, Project administration, Methodology, Funding acquisition. **Le Cai:** Writing – review & editing, Supervision, Resources, Project administration, Methodology, Funding acquisition.

## Funding information

The data collection and analysis of this study were supported by grants from the National Natural Science Fund of China (grant numbers: 72564027), Major Union Specific Project Foundation of Yunnan Provincial Science and Technology Department and Kunming Medical University (202401AY070001-027), the key research and development program of Yunnan Provincial Science and Technology Department (202403AC100015-03), and the First-Class Discipline Team of Kunming Medical University (2024XKTDTS16). The funders had no role in the study design, decision to publish, or preparation of the manuscript.

## Declaration of competing interest

The authors declare that they have no known competing financial interests or personal relationships that could have appeared to influence the work reported in this paper.

## Data Availability

Data will be made available on request.
